# Effects of a Standardized DBT—A Program on Identity Development in Adolescents

**DOI:** 10.3390/brainsci13091328

**Published:** 2023-09-15

**Authors:** Andrea Dixius, Eva Möhler

**Affiliations:** 1 Department of Clinical Pharmacy, Saarland University, 66123 Saarbrucken, Germany; eva.moehler@uks.eu; 2 SHG Clinic for Child and Adolescent Psychiatry, 66119 Saarbrucken, Germany; 3 Child and Adolescent Psychiatry, Saarland University Hospital, 66421 Homburg, Germany

**Keywords:** emotion regulation, identity, DBT-A, adolescents

## Abstract

Background: Identity diffusion plays a central role in the onset of borderline personality disorders. Dialectical Behavioral Therapy for Adolescents (DBT-A) is a treatment program for adolescents with emotional instability and dysregulation. The interest of this study is to examine the potential effects of a standardized and certified DBT-A therapy program on the identity development of adolescents in an inpatient setting. Methods: A total of 138 adolescents aged 13 to 18 years with symptoms of severe emotional instability were assessed before and after a certified and standardized 12-week in-patient DBT-A program targeting emotional regulation with the following standardized instruments: the Assessment of Identity Development in Adolescence (AIDA), Scale of the Experience of Emotions (SEE), and Symptom Checklist (SCL-90-R). Results: The results indicate a significant change in identity development, emotion regulation, and general symptoms of psychopathology after treatment with DBT-A. Conclusions: In this large sample of adolescents, DBT-A significantly improved identity development and reduced identity diffusion, however, without a treatment-as-usual control group as a limitation. Nevertheless, our results may become clinically relevant for the prevention of chronic impairment in emotionally unstable adolescents.

## 1. Introduction

During adolescence, numerous developmental tasks need to be achieved, including dealing with adaptive and maladaptive emotional regulation strategies. The possibility of diagnosing BPD in adolescence is considered reliable and valid [1]. Based on the assumption that adolescents with deficits in emotional regulation are a particularly vulnerable group for the development of mental disorders, early interventions are indicated to counteract the chronification of mental disorders [2].

With regard to the development of personality disorders, identity diffusion is understood to be an important factor in pathological development [3,4,5,6]; in fact, a central criterion of BPD is a disturbance of emotional regulation and identity development [7]. Identity development plays a central role in healthy and disturbed development from childhood [8,9], and identity diffusion is related to personality disorders [10], oftentimes associated with unstable attachments, experiences of invalidation, and negative childhood experiences [11]. Identity is described in DSM 5 as a core symptom (criterion 3) of borderline personality disorder [12,13,14]. Moreover, studies indicate a significant relationship between disturbed identity experience and the severity of psychopathology [3,15]. The sense of self is determined by coherence and continuity [3,8,16,17] and has a significant influence on the development of, e.g., autonomy [18].

Identity, according to [19], can be fundamentally defined, on one hand, by focusing on the self-referential aspect and, along with it, the idea of being unique as a person, more specifically to be a person with a past and future and be different from others. On the other hand, identity also includes the experience of being similar to others or sharing with others. Identity has also been reported to significantly impact autonomy development and implies comparison with others involving a dimension in the social context of “belonging” and “differentiation” [20]. Thus, identity might be considered in the social context, depending on social roles such as occupation, peer group, nationality, child, mother, father, friend, etc. The sense of self is determined by coherence and continuity [10,19]. 

With regard to identity development, there are no studies elucidating the factors contributing specifically to aspects of coherence and continuity and establishing predictors. However, it seems extremely important to assess influential factors, thereby identifying potential factors of prevention in child and adolescent mental health. 

The research results imply that stable identity development is of great importance for personality development and emotional development in adolescence [17]. Unstable identity development can significantly contribute to the development of personality disorders, in particular, borderline personality disorder (BPD) [21,22]. The detection of early antecedents of impaired identity development therefore seems an important step to allow for early intervention. In contrast to normal identity development, identity diffusion is seen as a lack of integration of the concept of the self and significant others, with a high risk of developing interpersonal problems, e.g., with family, school, peers. Identity diffusion contributes to pathological personality structures with a wide range of maladaptive and dysfunctional behaviors, accompanied by uncertainty, contradictions, a lack of skill of self-definition, and deficits in autonomy development.

Dialectical Behavioral Therapy (DBT) was developed by Marsha M. Linehan [23,24,25] as a disorder-specific therapeutic approach for the treatment of adults with borderline personality disorder (BPD). The effectiveness of DBT has been demonstrated in numerous studies [9,15,24,26,27]. A central criterion of BPD is the disturbance of emotional regulation, as well as disturbed cognitions and maladaptive behavior. An adaptation specifically designed for the treatment of adolescents (DBT-A) was developed by Miller and Rathus [7,12,28] (and a German version by Fleischhaker and colleagues [13]). Evidence-based data indicate the promising efficacy of DBT-A for adolescents with BPD symptoms, and its effectiveness has been demonstrated by numerous studies and reviews [7,9,14,28].

DBT-A can be applied to a variety of disorders by focusing on unstable emotional regulation [29]. In DBT-A, the goal is to convey adaptive skills to adolescents as alternatives to maladaptive behavior like self-injurious behavior, impulsive behavior, etc. through targeted skills training [29,30]. The interventions are characterized by a clear structure and a high practical relevance. The basic attitude is dialectical, validating, and mindful. In modules such as skills training, specific abilities are trained in the adolescents, e.g., to increase stress resilience, and regulation of strong inner states of tension and emotional regulation [30,31,32].

Many child and adolescent psychiatric disorders (e.g., eating disorders, borderline personality disorder, depression) are accompanied by—or even caused by—a clinically relevant impairment of identity development [15,33]. As with these findings, it is relevant to know whether central aspects of identity development in adolescents can be targeted effectively by a standardized DBT treatment program. Along with the assessment of a potential effect on identity, we investigate the related variables, emotion regulation, and general psychopathology, as these are targeted specifically by the DBT-A treatment program.

The present pilot study therefore examines the influence of a structured DBT-A program on identity development in adolescents diagnosed with BPD or severe emotional dysregulation, with the hypothesis that emotion regulation, psychopathology, and even a core trait as fundamental as identity development can be improved by this structured and certified treatment program.

## 2. Materials and Methods

### 2.1. Sample Description

A total of 188 adolescents were enrolled in the study, having been referred to inpatient treatment for emotional dysregulation such as emotional instability, self-harming behavior, or impulsive and aggressive behavior. 

The study inclusion procedure consisted of a session with a child and an adolescent psychiatrist evaluating admissibility regarding indication for elective inpatient treatment and delivering information about DBT-A and the group intervention, as well as informed consent. All the patients received a handout with information and worksheets at the beginning of the study. After informed consent, the adolescents were included in the treatment group. 

A total of 138 patients who completed the entire 12-week DBT treatment were finally included for analysis, consisting of 17 male and 121 female patients aged between 13 and 18 years (*M* = 16.5; *SD* = 1.3).

### 2.2. Inclusion and Exclusion Criteria 

Inclusion criteria:

- Aged 13–18;
- Inpatient referral for non-suicidal self-harming behavior, aggressive/impulsive behavior, and emotional crises;
- Voluntary participation in the structured 3-month DBT-A in-patient program.

Exclusion criteria:

- Diagnoses of schizophrenic or affective psychosis.

### 2.3. Diagnoses

Confirmation of the ICD-10 diagnoses (see Table 1) follows a clinical routine workflow with standardized diagnostic procedures specific to the disorder in question. Clinical interviews according to the symptom catalogs of ICD-10 were performed by either board-certified psychotherapists or consultant child and adolescent psychiatrists (medical doctors with a certified specialization).

Although diagnoses (ICD-10) were documented as presented in Table 2, they were not a prerequisite for participating in the study. 

### 2.4. Experimental Intervention 

DBT-A is a manualized program based on previous research. It comprises different modules within a 12-week time span. DBT-A was administered in single sessions twice a week, along with an additional session with a nurse for psychoeducation and skills training promotion. 

The skills group training took place twice a week for 60 min and was held by one therapist and one nurse. The group was conducted with 6–8 adolescents between the ages of 13 and 18. All the participants obtained worksheets for the treatment program. Overall, parents or key caregivers were offered facultative skills training (8 sessions).

Each module is structured and follows a standard sequence of activities and tasks, including tools for mindfulness, stress regulation, emotion regulation, interpersonal skills, and “walking the middle path”. In a short overview: Mindfulness is described as a specific form of attention, which differs from automatic everyday processes of perception. It is intentional, non-judgmental, and focused on consciously experiencing the current moment. Stress-tolerance exercises are learning to apply skills in high-stress phases and to deal with acute crises. Hence, this is about actively changing the pressuring situation that might arise. Emotion regulation: In the module “emotional regulation”, the adolescents receive low-threshold psychoeducation about the origins and functional purpose of feelings. A special focus of the module is the link between emotions and thoughts, behavior, perception, and physical reactions–emotional network. The module centered around self-esteem is focused on expanding social competences and self-acceptance in adolescents. Roleplay is used to convey strategies that can be used to build up and maintain self-esteem. Working with evaluation and dysfunctional cognitive schemes is the focus in this module and its respective exercises. Interpersonal skills: The perception and improvement of social competences are developed from roleplay and with specific examples. The application of helpful skills is thereby practiced. Aides for orientation are given to achieve certain goals: orientation towards personal goals (e.g., finishing school); orientation towards relationships (e.g., strengthening existing relationships); and orientation towards self-esteem (building up and maintaining). Walking the Middle Path: This module was specially designed for parental integration (alternatively, other legal guardians or reference persons of the adolescent) [7].

The middle path describes the work with dialectic views and skills that are practiced with both the adolescents and their reference people. 

The therapists need to undergo extensive training with additional certification provided by the DBT-A organization, including theoretical training and a standardized videotaped assessment of practical abilities, and conducting skills and mindfulness groups as well as individual therapy sessions.

### 2.5. Research Design 

Immediately before and after the standardized and certified DBT-A treatment was undertaken, the following assessments were performed by scientific personnel, different from the patients’ therapists to avoid suggestive effects.

### 2.6. Psychometric Assessments

The adolescents were asked to complete the following questionnaires, using a data-protected computer in a quiet setting, accompanied by a therapist in case of comprehension issues regarding the material or other questions.

#### 2.6.1. Assessment of Identity Development in Adolescence (AIDA) 

AIDA [19] (is a self-report questionnaire and a reliable and valid diagnostic instrument to detect disturbed identity development. This questionnaire distinguishes between identity diffusionand stable identity. 

In clinical practice, it supports the differentiation between normal and severely disturbed identity as the core component of BPD. 

An AIDA can serve as a tool for indication and evaluation of treatment methods that focus on identity. It takes into account influences from social–cognitive self-concept research. The main scales, “Continuity” and “Coherence”, consider emotionally intuitive and cognitive definition-related areas [3,17,20,34].

In clinical practice, it supports the differentiation between severely disturbed identity, as one of the core components of personality disorders like BPD, and identity crisis or stable identity development that can be found in other mental disorders. AIDA is developed as a self-screening instrument for adolescents between the age of 12–18 years. The goal is the differentiation between healthy identity development, a simple identity crisis, and clinically noticeable identity diffusion. 

#### 2.6.2. Reliability

Cronbach’s alpha was used to determine reliability, with 0.94 for the overall identity diffusion scale, 0.87 and 0.92 for the two main scales, discontinuity and incoherence, and between 0.69 and 0.84 for the subscale level. AIDA also showed excellent internal consistency for the total scale, “Identity Diffusion”, with Cronbach’s *α*: 0.94.

#### 2.6.3. Validity

The overall identity diffusion scale differentiated highly significantly and with relevant effect sizes between the school sample and the patient sample. The AIDA total score provides a very large effect size of *d* = 2.6 standard deviations.

#### 2.6.4. Depression Inventory for Children and Adolescents (DIKJ) [35]

This method is used to record the severity of depressive symptoms in children and adolescents aged 8 to 16 years. All the essential symptoms of a depressive disorder according to the criteria of the DSM-5 are being screened for. The DIKJ is also sensitive to changes in the severity of a depressive disorder. The internal consistency (Cronbach’s alpha) was found to be 0.92 in a clinical sample (N = 139) and 0.87 in an unscreened school sample (N = 3403).

#### 2.6.5. Symptom Checklist (SCL-90-R) [36]

SCL-90-R general psychopathology was measured via the German version of the revised Symptom Checklist, consisting of the scales, somatization, depression, compulsions, general anxiety, social anxiety, phobic anxiety, psychoticism, paranoia, hostility, and the global severity index as described by Grob and colleagues [36]. The level of symptomatic distress is recorded on a five-point Likert scale (“not at all”, “a little”, “fairly”, “strongly”, and “very strongly”). Three global parameters provide information about the overall level of psychological stress experienced.

This is a self-assessment procedure for adolescents and adults aged 12 and older. The internal consistency of the nine scales (Cronbach’s *α*) reached $r_{min}$ = 0.64 and $r_{max}$ = 0.84, as established in a sample of *n* = 857 adolescents aged 12–17 years. Three global characteristic values provide information about the overall level of psychological stress experienced. According to the author, the objectivity of implementation, evaluation, and interpretation is given. The content validity is assumed by the author on the basis of interviews with experts. 

#### 2.6.6. Questionnaire for the Assessment of Emotional Regulation in Children and Adolescents (FEEL-KJ) 

The FEEL-KJ questionnaire [37] is designed for children and adolescents aged 10 to 19, 11 years and quantifies 15 strategies for emotional regulation and regulation of specific emotions (all of which are multi-dimensional and specific to a certain emotion): anxiety, sadness, and anger. In two secondary scales, this instrument identifies seven adaptive and five maladaptive emotional regulation strategies. Adaptive strategies include the following sub-scales: problem-oriented acting, distraction, mood improvement, acceptance, forgetting, cognitive problem-solving, dealing with anger, anxiety, and grief. Maladaptive strategies include the sub-scales: giving up, aggressive behavior, self-devaluation, withdrawal, and perseveration, as well as dealing with anger, anxiety, and grief. The additional scales are composed of social support, expression, and emotional control. 

The internal consistency of the 15 scales ranges between *α* =0.69 and *α* = 0.91. The secondary scales show consistency of *α* = 0.93 (adaptive strategies) and α = 0.82 (maladaptive strategies). The range of the retest reliability (6-week stability) is between *r* = 0.62 and *r* = 0.81 and, for the secondary scales, between *r* = 0.81 (adaptive strategies) and *r* = 0.73 (maladaptive strategies). The secondary scale, called “adaptive scales” for specific emotions, shows a very good internal consistency, *α* = 0.88 for sadness, *α* = 0.83 for anxiety, and *α* = 0.83 for anger. The maladaptive scale shows internal consistency for anxiety *α* = 0.59, for sadness *α* = 0.59, and for anger *α* = 0.58. 

#### 2.6.7. Scales for Experiencing Emotions (SEE) 

The Emotional Experience Scales [38] are designed for use in individual and group examinations with adolescents aged 14 years and older, as well as adults. It is a theory-based, multidimensional measuring instrument for the central constructs of patient-centered personality theory and the concept of emotional intelligence. The questionnaire is divided into seven scales obtained through factor analysis and independent of each other. The scales contain 42 items, which include the following seven independent scales: 1. Acceptance of own emotions, 2. Experience of emotion transfer, 3. Experiencing emotional deficiency, 4. Body-related symbolization of emotions, 5. Imaginative symbolization of emotions, 6. Experiencing regulation of emotions, and 7. Experiencing self-control.

The scales aim to measure how patients perceive, evaluate, and address their emotions.

The internal consistency of the scales lies between 0.70 and 0.86 (Cronbach’s alpha). Additionally, retest reliability is reported to range from 0.60 to 0.090 between intervals of 2, 3, 4, 10, and 14 weeks.

### 2.7. Statistical Analyses

All the analyses were conducted using IBM Statistics SPSS, version 27.0.

A pre-intervention test was used to detect possible differences between completers and non-completers using a multivariate analysis of variance (MANOVA) with identity diffusion, general psychopathology, and emotional regulation as dependent variables. A multivariate analysis of variance (MANOVA) is used to test the statistical significance of the effect of one or more independent variables on a set of two or more dependent variables. 

Only data from the 138 participants who completed the DBT-A therapy program and answered all the questionnaires completely, thus called “completers”, were included in the analysis. Multivariate analysis of variance (MANOVA) was used to examine possible differences between completers and non-completers (*n* = 50) in the interval-scaled dependent variables of interest: identity (AIDA), general psychopathology (SCL-90-R), depression (DIKJ), and emotion regulation (FEEL-KJ, SEE). 

Dependent-sample *t*-tests were performed for within-group comparisons of pre- and post-measuring treatment outcomes. Cohen’s *d* (pooled effect size) was calculated to determine the effect size of the pre-/post-tests. For this purpose, Cohen recommends values of 0.20 for small effects, 0.50 for medium effects, and 0.80 for large effects [Statistical Power Analysis for the Behavioral Sciences (revised edition)].

Standardized norm values (t-values) were used for all the statistical analyses and descriptive plots.

Pearson’s correlation coefficients were calculated to determine the linear relationship between identity development scores, general psychopathology scores, and emotion regulation scores.

## 3. Results

Sample description (see Table 2): 17 male and 121 female patients completed the program, with a mean age of 16.5 years ranging from 13.7 to 18.8 years.

### 3.1. Comparison “Completer versus Non-Completer”

In the final analyses, only those patients (*n* = 138) were included who took part in the entire 12-week-long program (completers). To examine possible differences at the beginning of treatment between completers and those patients who did not complete the therapy program (non-completers), a multivariate analysis of variance (MANOVA) with the dependent variables’ identity diffusion, general psychopathology, and emotional regulation was carried out. 

In the multivariate tests, there were no statistically significant differences at the beginning of the therapy between completers (*n* = 138) and non-completers (*n* = 50) based on the dependent variables, *F*(7.18) = 1.62, *p* > 0.05, Wilk’s Λ = 0.94, and partial *η*^2^ = 0.059. However, the tests of the between-subjects effects and the individual contrasts revealed statistically significant differences (see Table 3) between the two groups on the following scales:

Identity Diffusion (AIDA): F(1) = 4.80, *p* < 0.05, *η*^2^ = 0.025;

Global Severity Index (GSI) (SCL-90-R): F(1) = 6.27, *p* < 0.05, *η*^2^ = 0.033.

This states that significantly higher values were measured on the reported scales in the group of completers at the start of therapy than in the non-completers (M and SD, see Table 3). There were no statistically significant differences between completers and non-completers at the start of therapy on the following scales:

Adaptive strategies (FEEL-KJ): F(1) = 2.82, *p* > 0.05, *η*^2^ = 0.015;

Maladaptive strategies (FEEL-KJ): F(1) = 2.28, *p* > 0.05, *η*^2^ = 0.012.

In sum, no significant differences in the reported scales were measured in the group of completers and non-completers at the start of therapy (M and SD, see Table 3).

#### 3.1.1. Assessment of Impairment of Identity Development in Adolescence (AIDA) [19] 

The main scale and two subscales of the AIDA were examined using repeated measures *t*-tests before and after the standardized DBT-A program (see Table 4).

The *t*-test for the diffusion subscale revealed a significant difference, *t*(137) = 5.58, *p* < 0.001, *d* = 0.67. Thus, higher values of identity impairment were measured in the patients before treatment (*M T*1 = 65.79, *SD T*1 = 13.13) than after the treatment (*M T*2 = 58.74, *SD T*2 = 15.92).

The *t*-test for the discontinuity subscale also revealed a significant result, *t*(137) = 5.10, *p* < 0.001, *d* = 0.63. Thus, higher values were measured in the patients before the hospital stay (*M T*1 = 67.20, *SD T*1 = 12.93) than after treatment (*M T*2 = 60.88, *SD T*2 = 15.45).

Finally, the *t*-test for the subscale, “incoherence”, also showed a significant difference, with *t*(137) = 5.00, *p* < 0.001, *d* = 0.68. Thus, higher impairment values were measured in the patients before the treatment program (*M T*1 = 63.17, *SD T*1 = 13.41) than after the treatment program (*M T*2 = 57.18, *SD T*2 = 15.25).

#### 3.1.2. Depression Inventory for Children and Adolescents (DIKJ) [35]

The *t*-test for the “severity of depressiveness” scale (see Table 4) revealed a significant result, *t*(137) = 7.09, *p* < 0.001, *d* = 0.91, indicating that patients had a higher “severity of depressiveness” score before treatment (*M T*1 = 67.82, *SD T*1 = 11.71) than after treatment (*M T*2 = 59.71, *SD T*2 = 14.68).

#### 3.1.3. Symptom Checklist (SCL-90-R) [36] 

The two scales measuring general psychopathology were tested with two repeated measures *t*-tests.

The *t*-test for the SCL-90-R scale (see Table 4) here for the Global Severity Index (GSI) also showed a significant result*, t*(137) = 7.28, *p* < 0.001, *d* = 0.83, indicating that patients had higher scores in total general psychopathology before treatment (*M T*1 = 64.18, *SD T*1 = 11.79) than after treatment (*M T*2 = 56.46, *SD T*2 = 14.59).

#### 3.1.4. Questionnaire for the Assessment of Emotional Regulation in Children and Adolescents (FEEL-KJ) [37]

The two main scales of the FEEL-KJ—“Adaptive Strategies” and “Maladaptive Strategies” were examined using two repeated measures *t*-tests.

**Figure 1:**
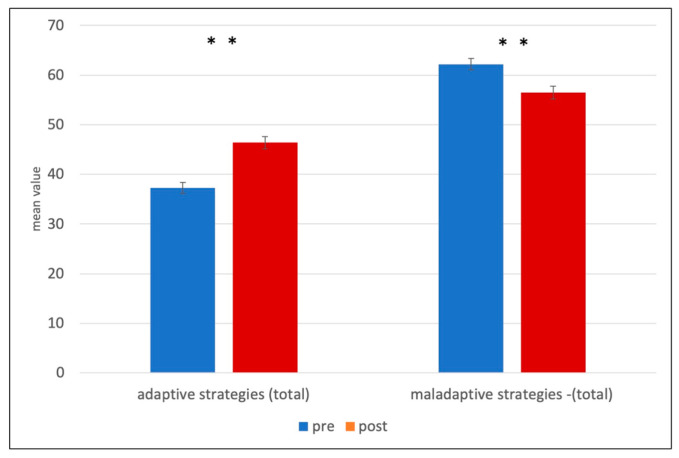
Questionnaire for the Assessment of Emotional Regulation in Children and Adolescents (FEEL-KJ) (Grob & Smolenski, 2009) adaptive and maladaptive scales in total—. ** *p* ≤ 0.001.

The *t*-test for the adaptive strategies scale shows a significant result in the pre-post comparison, *t*(137) = −7.26, *p* < 0.001, *d* = −1.02, indicating that patients had lower scores for the overall adaptive strategies scale before treatment (*M T*1 = 37.29, *SD* = 12.79) than after treatment (*M T2* = 46.41, *SD* = 14.45) (see Table 5, and Figure 1).

In addition, the *t*-test for the scale, maladaptive strategies, also revealed a significant result, *t*(137) = 4.81, *p* < 0.001, *d* = 0.48, indicating that patients had higher scores before DBT-A (*M T1* = 62.19 *SD T*1 = 13.42) than after DBT-A (*M T*2 = 56.47, *SD T*2 = 15.28).

The subscales were also examined with repeated measures *t*-tests.

Significant effects were also found with respect to the emotional regulation subscales for anger, anxiety, and sadness (see Table 5). There was a significant increase in scores for adaptive strategies and a significant reduction in scores for maladaptive strategies. Overall, small to moderate effects could be found (Cohen’s *d*) on the maladaptive strategies scales (*d* = 0.37 to *d* = 0.62) and large effect sizes (*d* = −0.80 to *d* = −1.03) for the adaptive strategies scale.

#### 3.1.5. Scales of Experience Emotions (SEE)

**Figure 2:**
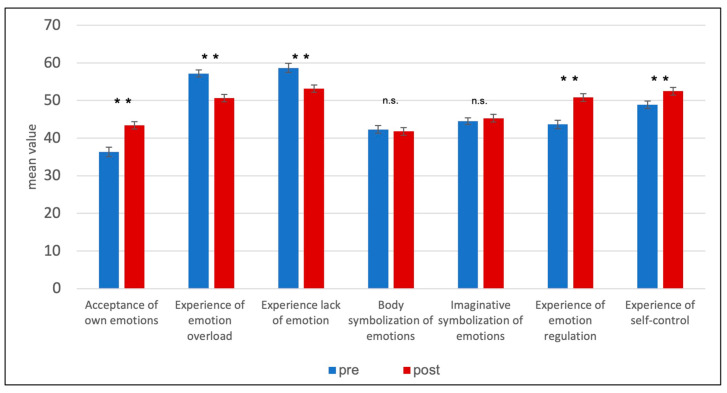
Scales of Experience Emotions (SEE). ** *p* ≤ 0.001; n.s. *p* > 0.05.

The scales of the SEE [38] were also examined using repeated measures *t*-tests before and after treatment with DBT-A. Significant results were found for the following scales (see Table 6; Figure 2).

Acceptance of own emotions: *t*(135) = −4.92, *p* < 0.001, *d* = −0.66.

This means that significantly lower values were measured before than after treatment:

Experience of emotional overload: *t*(135) = 5.62, *p* < 0.001, *d* = 0.82.

Experience of a lack of emotions: *t*(135) = 4.42, *p* < 0.001, *d* = 0.48.

Experience of emotion regulation: *t*(135) = −5.63, *p* < 0.001, *d* = −0.79.

Experience of self-control: *t*(135) = −3.88, *p* < 0.001, *d* = −0.46.

This means that significantly higher values (*M* and *SD*) were measured before than after treatment (Table 6).

There was no significant change on the following scales:

Body symbolization of emotions: *t*(135) = 0.41, *p* > 0.05;

Imaginative symbolization of emotions: *t*(135) = −1.02, *p* > 0.05.

This means that the values before and after the therapy do not differ significantly from each other (*M* and *SD*) (see Table 6).

## 4. Discussion

This pilot study examined whether Dialectical Behavioral Therapy for adolescent (DBT-A) inpatient treatment has an impact on the identity development of patients and on measures of psychopathology and emotional regulation in our 12-week-long certified structured and standardized DBT-A program. In the present study, it was of specific interest whether DBT-A could influence identity development as assessed by a validated and reliable measure with good internal consistency (AIDA). We were able to show an improvement in emotional regulation as reported by FEEL-KJ and SEE. Also, the patients significantly improved in measures of depression, as assessed by DIKJ, and general psychopathology, measured by SCL-90. These results are in line with previous findings on DBT-A [39,40].

The novel aspect of our study is that all the included patients appear to also benefit significantly from the standardized DBT-A in terms of their experience of identity, as indicated by the reduction of the identity diffusion values in the pre-/post- comparison of the pilot evaluation study. The Assessment of Impairment in Identity Development in Adolescence (AIDA) records a differentiated assessment of healthy and pathological identity development in adolescence [19,20]. These results are in line with studies reporting a general improvement in adolescent wellbeing and psychosocial functioning, as described by [39,40]; however, an effect of DBT-A on identity development as assessed by the AIDA questionnaire has never been reported, according to our review of the relevant literature. These novel findings are relevant and not only have a diagnostic value but also support the implementation of an early therapeutic intervention using DBT-A in unstable identity experiences to counteract further chronification in the course of the disease in adolescents [39,40].

These results also suggest the inclusion of a diagnostic assessment of identity development in emotionally unstable adolescents as a potentially important aspect of a full clinical diagnosis. The improvement in identity might hypothetically be caused by the increase in adaptive emotion regulation strategies and the reduction of maladaptive strategies, as a better capability of handling one’s emotions might be directly reflecting on an adolescent’s self-concept. A stable and positive self-concept is of essential importance to counteract potential chronification and disease progression fueled by a fundamentally negative self-concept [15,41,42]. The results therefore support an early therapeutic intervention using DBT-A in unstable identity experiences [41].

Numerous studies in the past have investigated correlations and risk factors for the development of severe emotion regulation disorders and the development of identity diffusion, yet studies on treatment approaches are lacking.

In the long term, early stabilization of adolescents is important, both in terms of counteracting the chronification of mental disorders and improvement of social integration and participation [2]. Emotional regulation and a stable identity can be regarded as central aspects of resilience [43]. In our study, as well as in DBT-A in general, the focus should be on symptomatology and levels of functioning and not exclusively on diagnosis [44].

These data cannot show changes in aspects such as body symbolization and imaginative symbolization by DBT-A; however, it can be hypothesized that in order to achieve changes in these deeply rooted characteristics, different psychotherapeutic approaches should probably be applied or added, such as the “Adolescent Identity Treatment” [45].

On a prospective note, especially in the field of adolescents, further studies on novel therapy approaches are needed to offer severely distressed adolescents therapies that validate their needs, especially in adapted methods, such as DBT-A, as a stepped-care treatment approach [20,46,47]. This could validate the needs of the adolescents as well as develop and offer customized therapy approaches regarding the necessary complexity and flexibility of the treatment (e.g., change between treatment settings—outpatient, partial inpatient, and online therapy modules). In particular, low-threshold therapy programs [2,14,45,48,49,50] promise to become important early and preventive interventions to counteract a chronification of impairment in disorders such as BPD, or PTSD [4] and other emotion regulation disorders.

### Limitations

The limitations of our study are the lack of a treatment-as-usual control group. Future studies in randomized control designs are underway. However, the proven feasibility and the large number of benefitting adolescents in this study give rise to the construction of a randomized control trial. Future studies including a treatment-as-usual group are underway.

## 5. Conclusions

The data presented in this article indicate an effect of DBT-A not only on emotion regulation and general psychopathology but also in identity development, thereby underlining the suitability of DBT-A for therapeutic intervention in adolescents with significant emotional instability.

## Figures and Tables

**Table 1:** Diagnoses—International Statistical Classification of Diseases (ICD-10).

Diagnosen	ICD-10	Frequency (*n*)	Percentage (%)
Adjustment disorder	F43.2	4	3
Anorexia nervosa	F50.1	25	18
Bulimia nervosa	F50.2	7	5
Dissociative convulsions	F44.5	1	1
Emotionally unstable personality disorder	F60.31	47	34
Moderate depressive episode	F32.1	15	11
Other mixed disorders of conduct and emotions	F92.8	1	1
Other specific personality disorders	F60.8	1	1
Post-traumatic stress disorder	F43.1	34	24
Social phobia	F40.1	2	1
Somatization disorder	F45.0	1	1

**Table 2:** Sample description.

Sample Description	
Participants (*n*)	*n* = 138
Gender % (*n*)	
Male	12.3 (17)
Female	87.7 (121)
*M* (*SD*)	16.5 (1.3)
Age (min.–max.)	13.7–18.8
*M* (*SD*)	14.9 (5.9)
(Duration of treatment in weeks)	

**Table 3:** Differences in completers vs. non-completers at the beginning of therapy.

	Completer *n* = 138		Non-Completer *n* = 50		MANOVA		
	*Mean T*1	*SD T*1	*Mean T*2	*SD T*2	*F(df)*	*Sig.*	Partial *η*^2^
Identity Diffusion (AIDA)	65.79	13.13	60.92	14.35	4.80 (1)	0.030	0.025
Depression (DIKJ)	64.18	11.77	57.78	13.93	9.82 (1)	0.002	0.050
GSI (SCL-90-R)	67.82	11.71	62.88	12.59	6.27 (1)	0.013	0.033
FEEL-KJ—adaptive strategies (total)	37.29	12.79	40.76	11.70	2.82 (1)	0.095	0.015
FEEL-KJ—maladaptive strategies (total)	62.19	13.42	58.80	14.05	2.28 (1)	0.133	0.012

AIDA: Assessment of Identity Development in Adolescence; FEEL-KJ: Questionnaire for the assessment of the emotional regulation in children and adolescents; GSI: Global Severity Index; SEE: Scales of Experience Emotions; SCL-90-R Symptom Checklist. Completer: patients who took part in the entire 12-week-long program; non-completer: patients who did not complete the 12-week program; M dif: mean value difference; T1: pre-treatment; T2: post-treatment; SD: standard deviation; partial *η*^2^: partial eta squared.

**Table 4:** Results: Pre-/Post- Comparison.

	*Mean T*1	*SD T*1	*Mean T*2	*SD T*2	*Mean dif*	*SD dif*	*t*	*df*	*p*	*d*
Diffusion (AIDA)	65.79	13.13	58.74	15.92	7.05	14.85	5.58	137	<0.001	0.67
Discontinuity (AIDA)	67.20	12.93	60.88	15.45	6.33	14.56	5.10	137	<0.001	0.63
Incoherence (AIDA)	63.17	13.41	57.18	15.25	5.99	14.09	5.00	137	<0.001	0.68
DIKJ	67.82	11.71	59.71	14.68	8.11	13.44	7.09	137	<0.001	0.91
GSI (SCL-90-R)	64.18	11.79	55.46	14.59	8.72	14.08	7.28	137	<0.001	0.83

Assessment of Identity Development in Adolescence (AIDA); Depression Inventory for Children and Adolescents (DIKJ); Symptom Checklist- (SCL-90-R: Global Severity Index).

**Table 5:** Questionnaire for the Assessment of Emotional Regulation in Children and Adolescents (FEEL-KJ).

FEEL-KJ	*Mean T*1	*SD T*1	*Mean T*2	*SD T*2	*Mean dif*	*SD dif*	*t*	*df*	*p*	*d*
Adaptive strategies (total)	37.29	12.79	46.41	14.45	−9.12	14.76	−7.26	137	<0.001	−1.02
Anger	38.11	12.09	46.66	13.50	−8.55	14.25	−7.05	137	<0.001	−1.03
Anxiety	37.04	11.96	46.93	14.11	−9.89	14.40	−6.14	137	<0.001	−0.80
Sadness	38.91	12.27	46.63	13.78	−7.73	14.52	−6.25	137	<0.001	−0.86
Maladaptive strategies (total)	62.19	13.42	56.47	15.28	5.72	13.96	4.81	137	<0.001	0.48
Anger	59.88	13.01	55.25	14.19	4.62	13.01	4.18	137	<0.001	0.49
Anxiety	60.51	13.72	55.78	15.33	4.73	15.23	3.64	137	<0.001	0.37
Sadness	61.43	12.17	55.56	13.73	5.87	12.88	5.35	137	<0.001	0.62

**Table 6:** Scales of Experience Emotions (SEE).

SEE	*Mean T*1	*SD T*1	*Mean T*1	*SD T*2	*Mean dif*	*SD dif*	*t*	*df*	*p*	*d*
Acceptance of own emotions	36.07	14.81	43.41	17.17	−7.34	17.42	−4.92	137	<0.001	−0.66
Experience of emotion overload	57.27	11.17	50.63	12.40	6.64	13.78	5.62	137	<0.001	0.82
Experience lack of emotion	58.94	14.08	53.17	13.23	5.77	15.23	4.42	137	<0.001	0.48
Body symbolization of emotions	42.15	11.79	41.79	11.76	0.36	10.27	0.41	137	>0.05	−
Imaginative symbolization of emotions	44.45	10.43	45.29	10.41	−0.84	9.61	−1.02	137	>0.05	−
Experience of emotion regulation	43.34	12.67	50.80	13.42	−7.46	15.95	−5.46	137	<0.001	−0.79
Experience of self-control	48.71	11.61	52.46	11.34	−3.74	11.24	−3.88	137	< 0.001	−0.46

*T*1: pre-treatment; *T*2: post-treatment; *SD*: standard deviation; *Mean dif*: mean value difference; *SD dif*: standard deviation difference; *d*: Cohen’s *d*.

## Data Availability

The data set can be obtained from the first author upon request andrea.dixius@uni-saarland.de.

## Abbreviations

AIDA: Assessment of Identity Development in Adolescence; BDP, Borderline Personality Disorder; DBT, Dialectical Behavior Therapy; DBT-A, Dialectical Behavior Therapy for Adolescents; DIKJ, Depression inventory for Children and Adolescents; FEEL-KJ, Fragebogen zur Erhebung der Emotionsregulation bei Kindern und Jugendlichen (Questionnaire for the assessment of the emotional regulation in children and adolescents); GSI, Global Severity Index; ICD, International Statistical Classification of Diseases; SEE, Scales of Experience Emotions; SCL-90-R, Symptom Checklist.
